# Resistant dextrin improves high-fat-high-fructose diet induced insulin resistance

**DOI:** 10.1186/s12986-020-00450-2

**Published:** 2020-05-15

**Authors:** Fan Hu, Yixin Niu, Xiaoyuan Xu, Qiuyue Hu, Qing Su, Hongmei Zhang

**Affiliations:** Department of Endocrinology, Xinhua Hospital, Shanghai Jiaotong University School of Medicine, Shanghai, China

**Keywords:** Dietary fiber, Resistant dextrin, Insulin resistance, Fatty acid beta oxidation, Gut microbiota

## Abstract

**Background:**

Insulin resistance is an important defect associated with obesity and type 2 diabetes mellitus. Many studies have been reported that dietary fiber exerts beneficial metabolic effects. Resistant dextrin is a soluble fiber. The aim of this study was to investigate the effects of resistant dextrin on high-fat-high-fructose diet induced obese mice and to explore the underlying mechanisms.

**Methods:**

Seventeen 4-week-old male C57BL/6 J mice were fed a normal diet (ND) or HFHFD for 22 weeks, and were gavaged with resistant dextrin (5 g/kg) for 10 weeks. Glucose tolerance test (GTT) and insulin tolerance test (ITT) were performed, serum fasting insulin (FINS) and serum biochemical parameters were determined, the contents of triglyceride (TG) and total cholesterol (TC) in liver tissues were determined by enzymatic method. The pathological changes in liver were detected by HE staining. Real time PCR and Western blot were used to detect the expression of insulin signaling pathway and the fatty acid β oxidation pathway related genes and proteins respectively. The gut microbiota were analyzed via 16 s rRNA sequencing.

**Results:**

Resistant dextrin significantly decreased serum FINS, improved serum lipid profiles, reduced the contents of liver TG and TC. The insulin signaling pathway and the fatty acid β oxidation pathway were promoted. The abundance of metabolically beneficial bacteria such as Prevotella and Akkermansia in the intestinal flora of the resistant dextrin group were increased.

**Conclusions:**

Resistant dextrin can significantly ameliorate liver insulin resistance, improve serum lipid levels, as well as reduce hepatic lipid deposition. The modulation of gut microbiota might be responsible for the beneficial effects of resistant dextrin.

## Background

In recent decades, the incidence and prevalence of type 2 diabetes mellitus (T2DM) has been dramatically increased, contributing great burden to global health economies [1]. Insulin resistance (IR) is an important defect associated with obesity and T2DM, which is defined as ‘a relative impairment in the ability of insulin to exert its effects on glucose and lipid metabolism in target tissues’ [2]. Improving insulin sensitivity is an available strategy for the management of T2DM.

More and more studies have been reported that dietary fiber exerts many beneficial metabolic effects, including improvement of IR and lowing the risk of developing T2DM. A recent meta-analysis reported that higher intakes of fiber are associated with a reduced risk of diabetes [3]. The effects are associated with decreased gastric emptying, reduced blood cholesterol levels, enhanced production of short chain fatty acids (SCFAs) and modulated the composition of the gut microbiota.

Though substantial evidence has shown the beneficial effect of dietary fiber for health, there is still a gap between recommendations and intake worldwide, due the fact that it is difficult to get enough fiber from a traditional diet. Therefore, supplementing the extracts derived from fruits and vegetables or synthetic non-digestible carbohydrates may be an effective way to increase dietary fiber intake [4]. Various fiber subtypes such as oligosaccharides, inulin, β-glucan and pectin are well-studied. However, because of the production of gases, they often induce intestinal discomfort even at small dosage. Compare to other fibers, resistant dextrin showed better tolerance [5–8]. Resistant dextrin is a soluble fiber, derived from wheat or corn starch and is prepared by highly controlled partial hydrolysis and repolymerization of the dextrinization process. Animal studies showed that resistant dextrin could stimulate gut mucosal immunity and prevent colitis in piglets [9]. Clinical trials demonstrated that supplementation with resistant dextrin for 12 weeks alleviates insulin resistance and improves determinants of metabolic syndrome in overweight men [10]. In addition, a daily supplement of 10 g resistant dextrin improves insulin resistance and inflammation in women with type 2 diabetes [11].

Although a few clinical studies have reported that resistant dextrin exerts beneficial effects on insulin resistance and T2DM, the evidence is insufficient and the mechanisms have not yet been elucidated. The aim of this study was to investigate the effects of resistant dextrin on HFHFD induced obese mice, especially to observe its effects on insulin sensitivity and lipid metabolism, and to explore the underlying mechanisms.

## Methods

### Animals and treatment

Four-week-old male C57BL/6 mice were purchased from Shanghai slac corporation. The mice were housed under a constant temperature (22 ± 2 °C) and 12-h light/dark cycle and maintained on a normal diet with clean water ad libitum for 1 week. Mice were randomly divided into two groups and fed a normal diet (ND,10% fat, *n* = 5) or high-fat-high-fructose-diet (HFHFD, 35.5% fat, *n* = 12) for 12 weeks. Then the HFHFD group were randomly divided into the high-fat-high-fructose diet plus resistant dextrin (HFHFD+RD) group and the HFHFD group, with six mice in each group. The mice in the two groups were gavaged with resistant dextrin 5 g/kg body weight or distilled water respectively for 10 weeks. Both groups continued the HFHFD. The ND group were still on a normal diet, and they were gavaged with distilled water for 10 weeks. During the intervention, food intake was recorded, body weight and fasting blood glucose (FBG) were measured.

### Glucose and insulin tolerance tests

Glucose tolerance test (GTT) was performed at the end of the intervention, mice were fasted 16 h. After measuring the baseline blood glucose level via a tail nick using a glucometer (Contour TS, Bayer, Germany), 2 g/kg glucose was administered via gavage, and glucose levels were measured at different time points of 15, 30, 60 and 120 min after glucose administration.1 week later, insulin tolerance test (ITT) was performed. Mice were fasted 6 h and they were injected intraperitoneally with recombinant human insulin (Novo Nordisk, Denmark) at 1 U/kg and their blood glucose concentrations were measured 0, 15, 30, 60 and 120 min after insulin injection.

### Sampling

Blood was collected after mice were sacrificed, centrifuged at 3000 rpm for 15 min at 4 °C, and stored at − 80 °C before serum profile analysis. The liver tissues were removed, weighted, frozen in liquid nitrogen, and stored at − 80 °C.

### Blood parameters

Serum FINS levels were detected using ELISA kit (EZRMI-13 K, Merckmilipore). HOMA-IR was calculated according to the formula FBG (mmol/L)*FINS (μU/L)/22.5. Serum TG, TC, Low-density lipoprotein cholesterol (LDL-Ch) and High-density lipoprotein cholesterol (HDL-Ch) levels were determined by automatic biochemical analyzer (ROCHE COBASc702).

### Hepatic TG and total TC content

Hepatic TG and total TC content were determined using a commercially available kit (GPO-POD, Applygen Technologies Inc., Beijing, China) according to the manufacturer’s protocols.

### Hematoxylin-eosin (H&E) staining

Take fresh liver tissue samples fixed, dehydrated and embedded in paraffin. After that, 4–6 μm serially sections were taken on a tissue microtome for routine HE staining, and pathological changes of liver tissue were observed under microscope.

### Quantitative real-time PCR analysis

Total RNA was extracted using Trizol Reagent (Invitrogen). cDNA was synthesized from total RNA using the kit (Takara) according to the manufacturer’s instructions. Quantitative RT-PCR was carried out in triplicate using a real time PCR system (Applied Biosystems). The β-actin gene was used as an endogenous control, for normalization of gene expression levels. The relative gene expression levels were assessed by using the 2^-ΔΔCt^ method. The primer sequences are listed in Table 1.

**Table 1:** The primer sequences

Primer	Sequences (5′ to 3′)
β-actin-F	GTGCTATGTTGCTCTAGACTTCG
β-actin-R	ATGCCACAGGATTCCATACC
PPARα-F	CACGCATGTGAAGGCTGTAA
PPARα-R	GCTCCGATCACACTTGTCG
CPT1A-F	AACCCAGTGCCTTAACGATG
CPT1A-R	GAACTGGTGGCCAATGAGAT
FXR-F	TTCCTCAAGTTCAGCCACAG
FXR-R	TCGCCTGAGTTCATAGATGC
ACOX-F	TAACTTCCTCACTCGAAGCCA
ACOX-R	AGTTCCATGACCCATCTCTGTC

### Western blot analysis

Liver tissue were lysed in RIPA buffer containing a protease inhibitor (Beyotime Institute of Biotechnology, China). 30 μg of total protein were separated using a sodium dodecyl sulfate-polyacrylamide gel electrophoresis (SDS-PAGE) and followed by the transfer of proteins to a PVDF membrane. After blocking with 5% skim milk, membranes were incubated with the appropriate primary antibodies overnight at 4 °C. Primary antibodies including anti-phospho-IRS-1^Ser612^(CST), anti-IRS-1(CST), anti-phospho-AKT^Ser473^(CST), anti-AKT (CST), anti-Glut-2 (Abclonal), anti-Sirt1(Abcam), anti-PPARα (Abclonal), anti-CPT1α(Abcam), anti-AMPK (Abcam). Blots were incubated with an appropriate horseradish conjugated secondary antibody (Beyotime Institute of Biotechnology, China) at room temperature for 1 h. Antibody bound protein was detected by ECL (Merkmillipore) and quantified by scanning densitometry. Relative protein expression was normalized to Tublin expression.

### Gut microbiota composition

The 16 s rRNA analysis of the fecal samples was performed by OE Biotech Co., Ltd. (Shanghai, China). Fecal samples were used for DNA extraction with the QIAxtractor (QIAGEN). The V3–V4 region of the 16 s rRNA gene was amplified. The original double-ended sequence was de-interleaved using Trimmomatic software, and the de-duplexed double-ended sequence was spliced using FLASH software. Using of UCHIME to detect and remove chimeric sequences. After the sequencing data was preprocessed to generate high-quality sequences, the Vsearch software was used to classify the sequences into multiple OTUs based on the similarity of the sequences. A parameter with a sequence similarity greater than or equal to 97% is classified as an OTU unit. Use the QIIME package to pick out the representative sequences of the individual OTUs, and compare all the representative sequences to the database, using the Silva (version123) database alignment. The PCA principal component analysis was used to analyze the differences between the samples. Using linear discriminant analysis effect size (LEfSe), linear discriminant analysis (LDA) was used to determine the groups with significant differences in genus or higher taxonomic levels between groups of mouse populations.

### Statistical analysis

For statistical analysis, we used the SPSS Statistical Package (version 22.0, SPSS Inc., Chicago, IL). Data followed normal distribution were presented as mean ± SD, and were analyzed by One-way ANOVA. Data which were not normally distributed were presented as median with interquartile range and were analyzed by Kruskal-Wallis test. *P* values< 0.05 were considered statistically significant.

## Results

### Effects of resistant dextrin on body weight and food intake

**Fig. 1:**
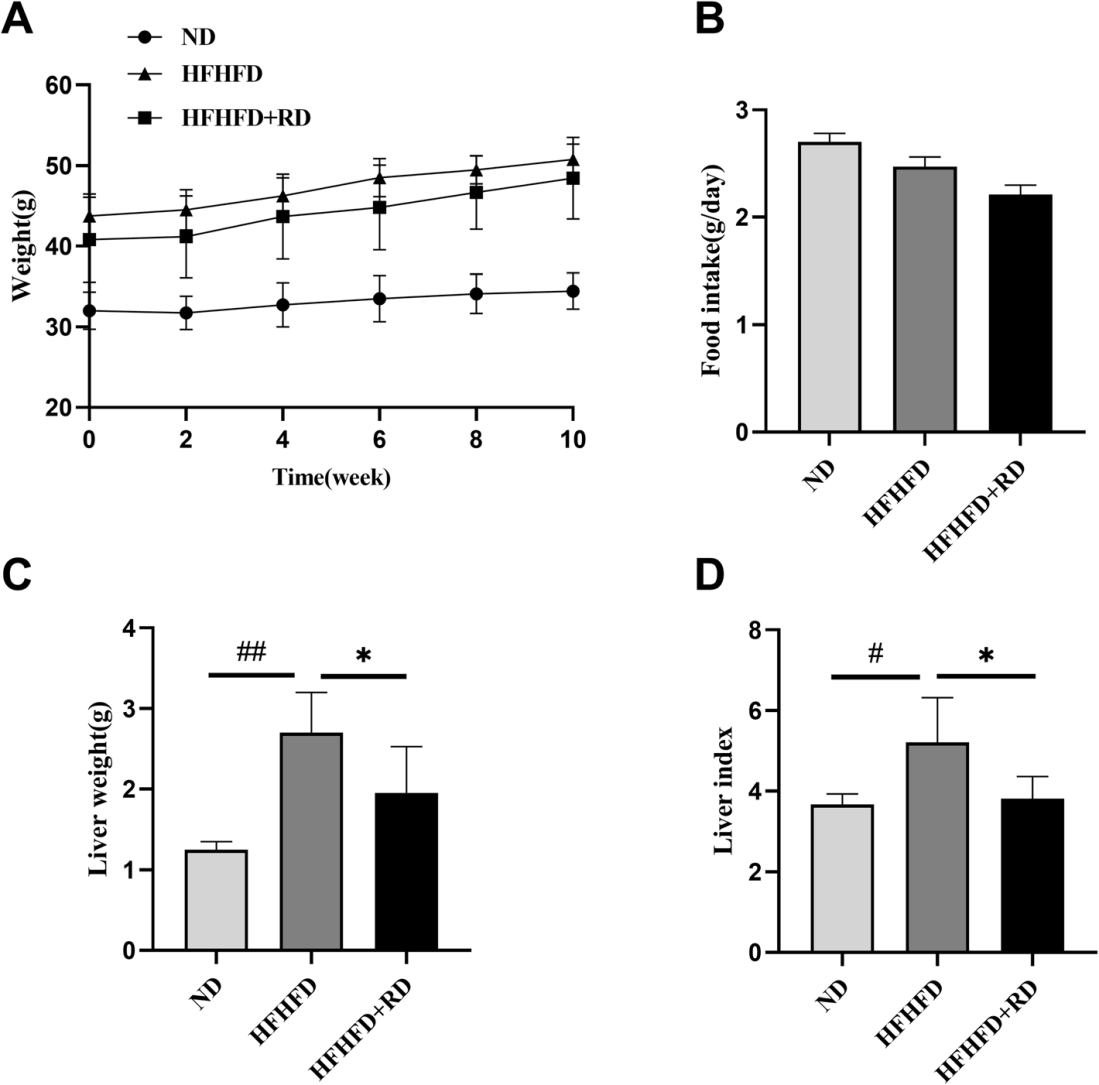
Effects of resistant dextrin (RD) on body weight and food intake. Body weight and food intake during the intervention of 10 weeks in the normal diet (ND) mice, HFHFD mice and HFHFD plus RD (HFHFD+RD) mice (**a** and **b**). Liver weight and liver index at the end of the intervention (**c** and **d**). Results were expressed as mean ± standard deviation. *P<0.05 vs. HFHFD group, #*P*<0.05, ##*P*<0.001 vs. ND group

After 12 weeks, the HFHFD-fed mice weighted significantly (*P*<0.001, Fig.S1A), and the level of FBG were also significantly increased (*P*<0.001, Fig.S1B), indicating that the obesity mice model was successfully established. After the supplement of resistant dextrin for 10 weeks, HFHFD+RD mice gained progressively less body weight compared with HFHFD mice, under the circumstances of no food intake changing (Fig. 1a-b). As for the liver weight, data showed a decreased liver weight and a reduced liver to body weight ratio in HFHFD+RD mice when compared with HFHFD mice (*P*<0.05, Fig. 1c-d).

### Resistant dextrin ameliorated insulin resistance induced by HFHFD feeding in mice and enhanced insulin signaling pathway in the liver

**Fig. 2:**
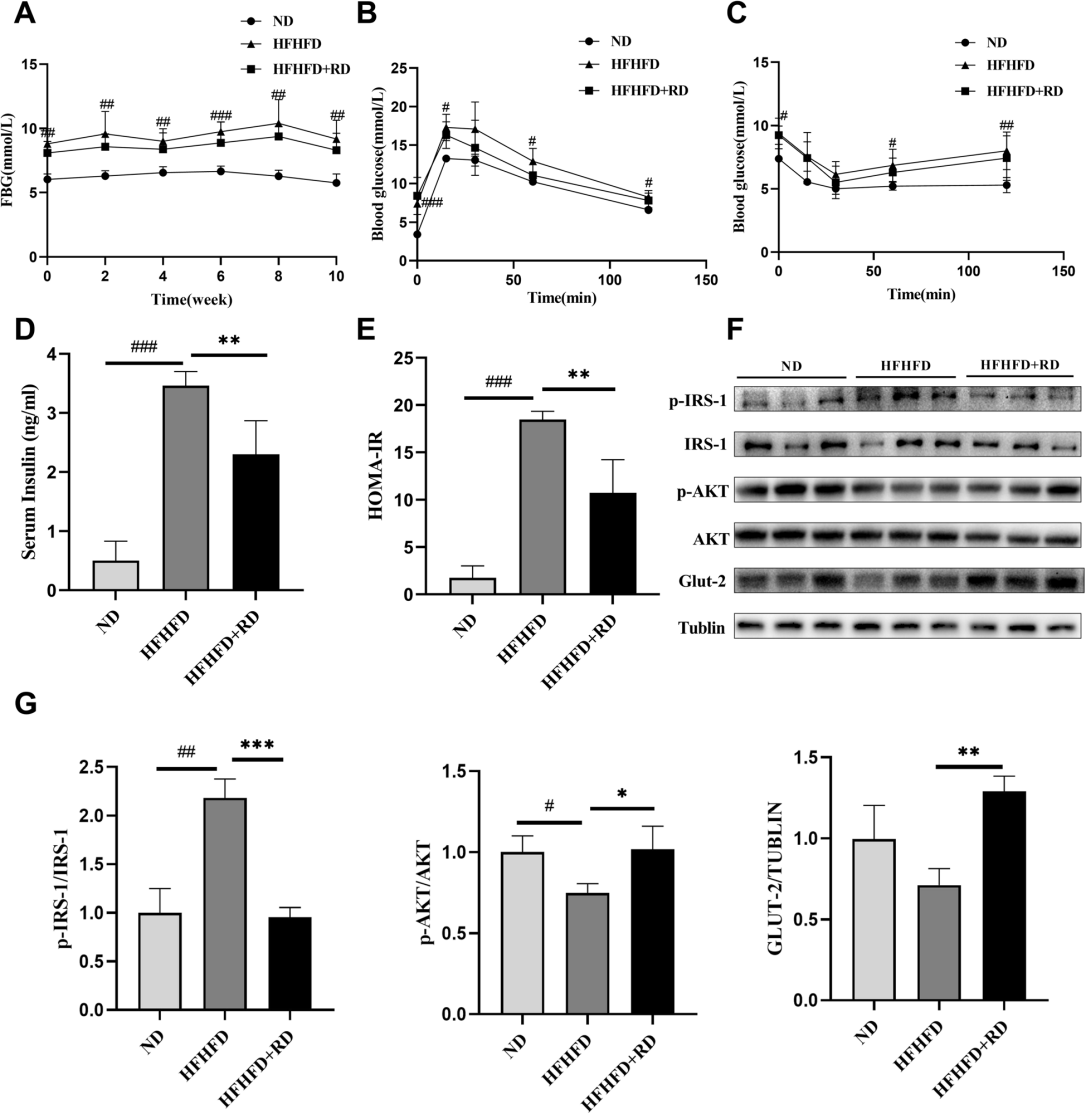
Resistant dextrin ameliorated insulin resistance induced by HFHFD feeding in mice. **a** The level of FBG during the intervention. **b** Oral glucose tolerance test (OGTT) was performed at the end of the intervention. **c** Insulin tolerance test was performed at the end of the intervention. **d** Changes in fasting serum insulin of three groups. **e** Changes in HOMA-IR of three groups. **f** Western blot assay of insulin signaling pathway related proteins in the liver. **g** Relative levels of insulin signaling pathway related proteins of p-IRS-1, p-AKT and GLUT-2 were determined by normalizing protein expressions versus IRS-1, AKT and tublin expression. Results were expressed as mean ± standard deviation. *P<0.05, **P<0.01 and ***P<0.001 vs. HFHFD group. #*P*<0.05, ##*P*<0.01 and ###*P*<0.001 vs. ND group

We measured FBG during the intervention and found that the levels of FBG in the HFHFD group was significantly higher than that in the ND group, however, the supplementation of resistant dextrin reduced the FBG levels induced by HFHFD (Fig. 2a). Similarly, the oral glucose tolerance test (OGTT) and insulin tolerance test (ITT) showed a significant higher blood glucose in the HFHFD group compared to the ND group, while a decreasing trend in the HFHFD+RD group though there was no significant difference (Fig. 2b-c). In addition, serum insulin levels and HOMA-IR were significantly decreased after the supplement of resistant dextrin (Fig. 2d-e). As shown in Fig. 2f-g, the levels of insulin signaling pathway related proteins p-IRS-1^ser612^/IRS-1 were remarkably increased, p-AKT^ser473^/AKT and GLUT-2 proteins were notably decreased in HFHFD group. Supplementation of resistant dextrin decreased the level of p-IRS-1^ser612^/IRS-1 and increased p-AKT^ser473^/AKT and GLUT-2 protein levels.

### Resistant dextrin improved serum lipid levels and reduced hepatic lipid deposition

**Fig. 3:**
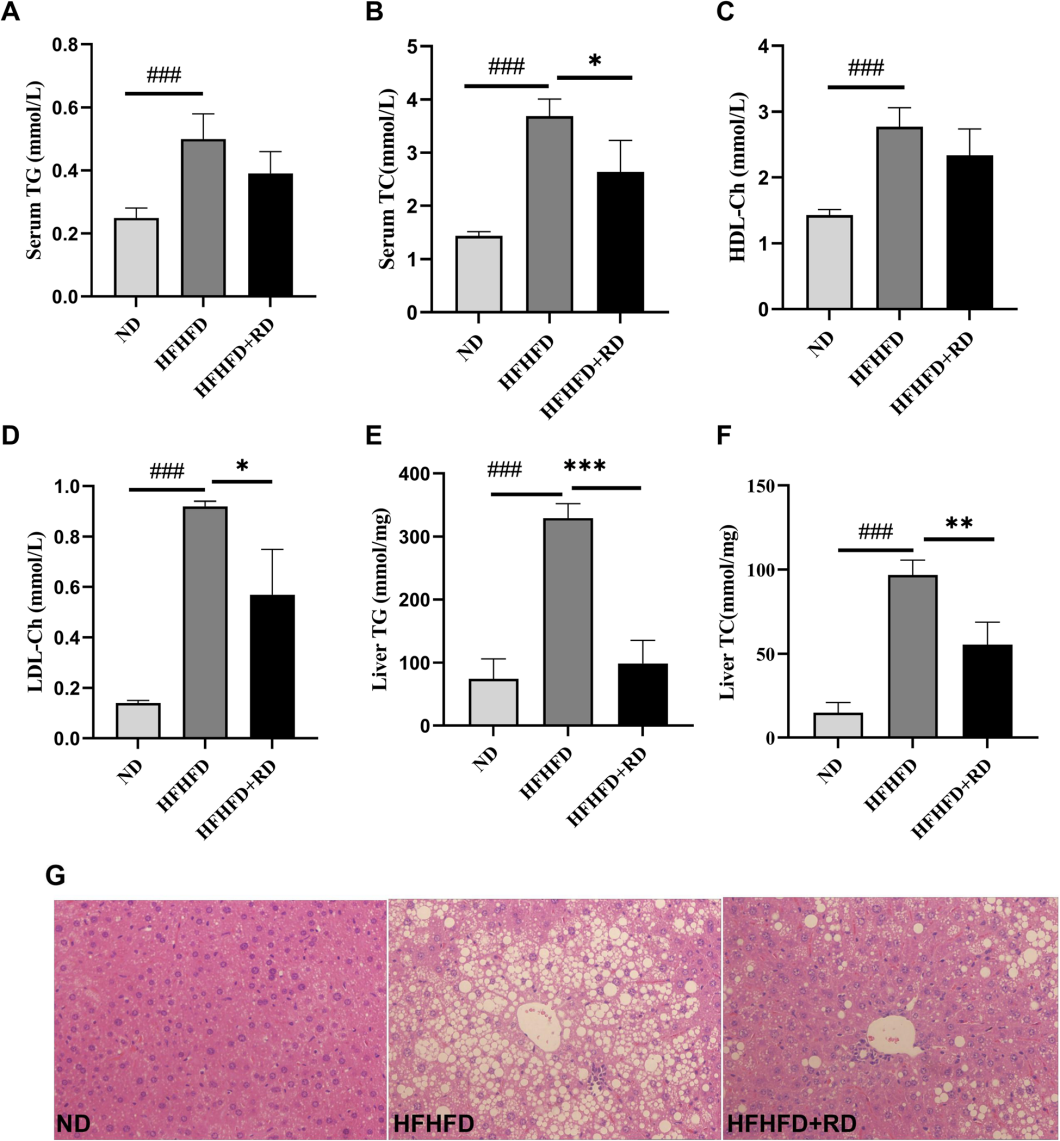
Resistant dextrin improved serum lipid levels and reduced hepatic lipid deposition. **a** Serum TAG, **b** serum TC, **c** serum HDL-Ch, **d** serum LDL-Ch levels in three groups. **e** Hepatic TAG and **f** total TC contents. **g** H&E staining of liver morphology in three groups (200× magnification). Results were expressed as mean ± standard deviation. *P<0.05,**P<0.01 and ***P<0.001 vs. HFHFD group. ###*P*<0.001 vs. ND group

Serum TG, TC, LDL-Ch and HDL-Ch were significantly higher in the HFHFD group, and 10 weeks supplement of resistant dextrin reduced serum TC and LDL-Ch significantly, indicating that resistant dextrin improved blood lipid levels (Fig. 3a-d). The results of liver TG and TC contents demonstrated that resistant dextrin reduced hepatic lipid deposition, as the contents of TG and total TC were significantly reduced compared with the HFHFD group (Fig. 3e-f). The results of H&E staining (Fig. 3g) further revealed that the sizes and numbers of hepatic lipid droplets were decreased and fatty degeneration was alleviated in the HFHFD+RD group. This was consistent with the results of hepatic TG and TC contents.

### Resistant dextrin promoted fatty acid β oxidation in the liver

**Fig. 4:**
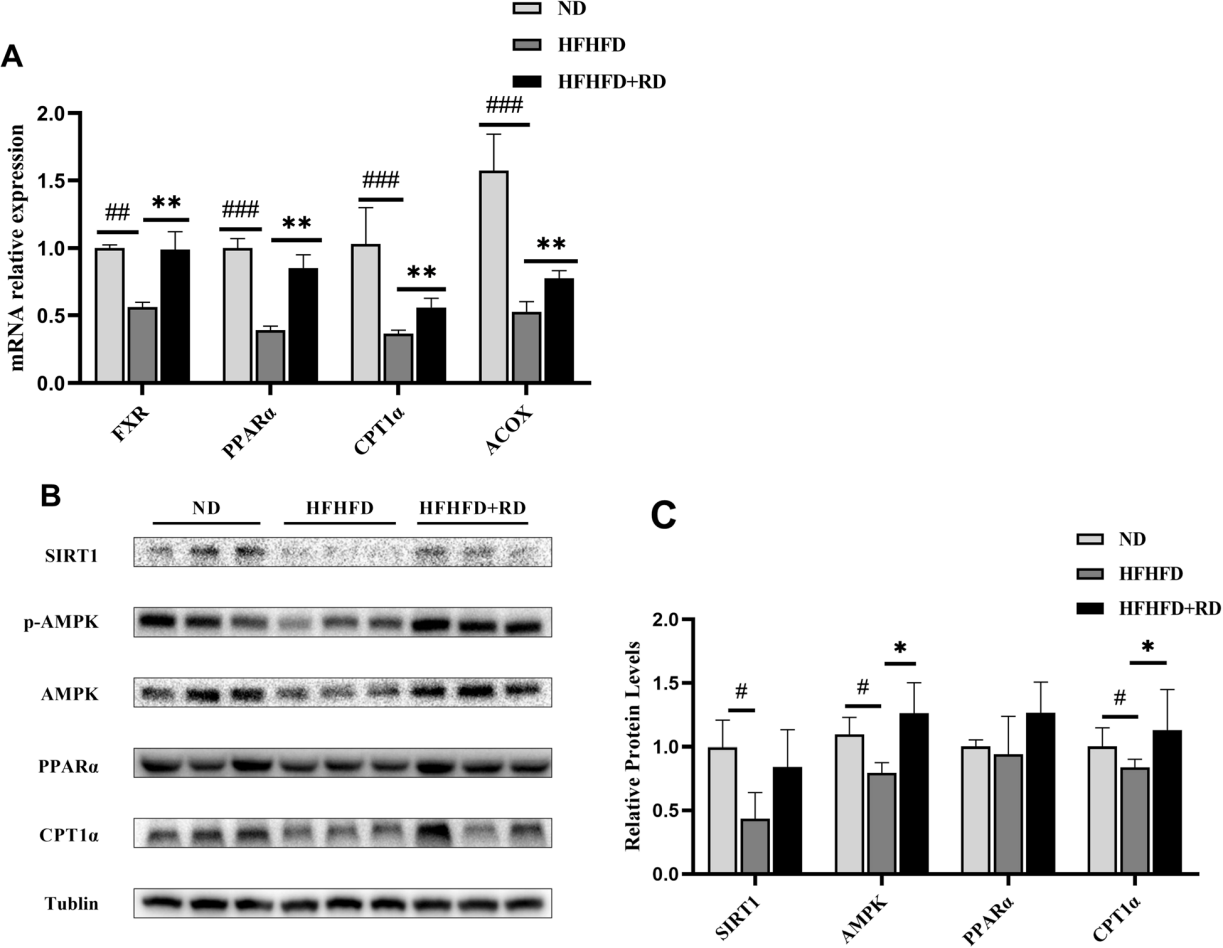
Resistant dextrin promoted fatty acid β oxidation in the liver. **a** qRT-PCR analysis of genes involved in fatty acid metabolism. **b** Western blot assay of fatty acid β oxidation related proteins in the liver. **c** Relative levels of β oxidation related proteins of Sirt1, AMPK, PPARα and CPT1α were determined by normalizing protein expressions versus tublin expression. Results were expressed as mean ± standard deviation. *P<0.05 and **P<0.01 vs. HFHFD group. #*P*<0.05 vs. ND group

Relative to mice in the HFHFD group, mice in the HFHFD+RD group showed significantly changes in the expression of FXR, PPARα, CPT1α and ACOX genes, which are involved in fatty acid oxidation (Fig. 4a). We also analyzed the expression of proteins involved in β-oxidation by western blotting. As shown in Fig. 4b-c, expression level of AMPK was significantly increased in HFHFD+RD group compared with the HFHFD group. Moreover, expression levels of Sirt1, PPARα and its target protein CPT1α were higher than that in the HFHFD group.

### Gut microbiota composition analysis

Results from UniFrac-based principal coordinates analysis (PCoA) revealed a distinct clustering of microbiota composition for each group (Fig.S2). LEfSe analysis was carried out to identify discriminative features. The HFHFD+RD group was characterized by a higher amount of Alloprevotella and Prevotellaceae family, which is consistent with the results of ND group, while the HFHFD group was enriched by Bacteriodes genus.

**Fig. 5:**
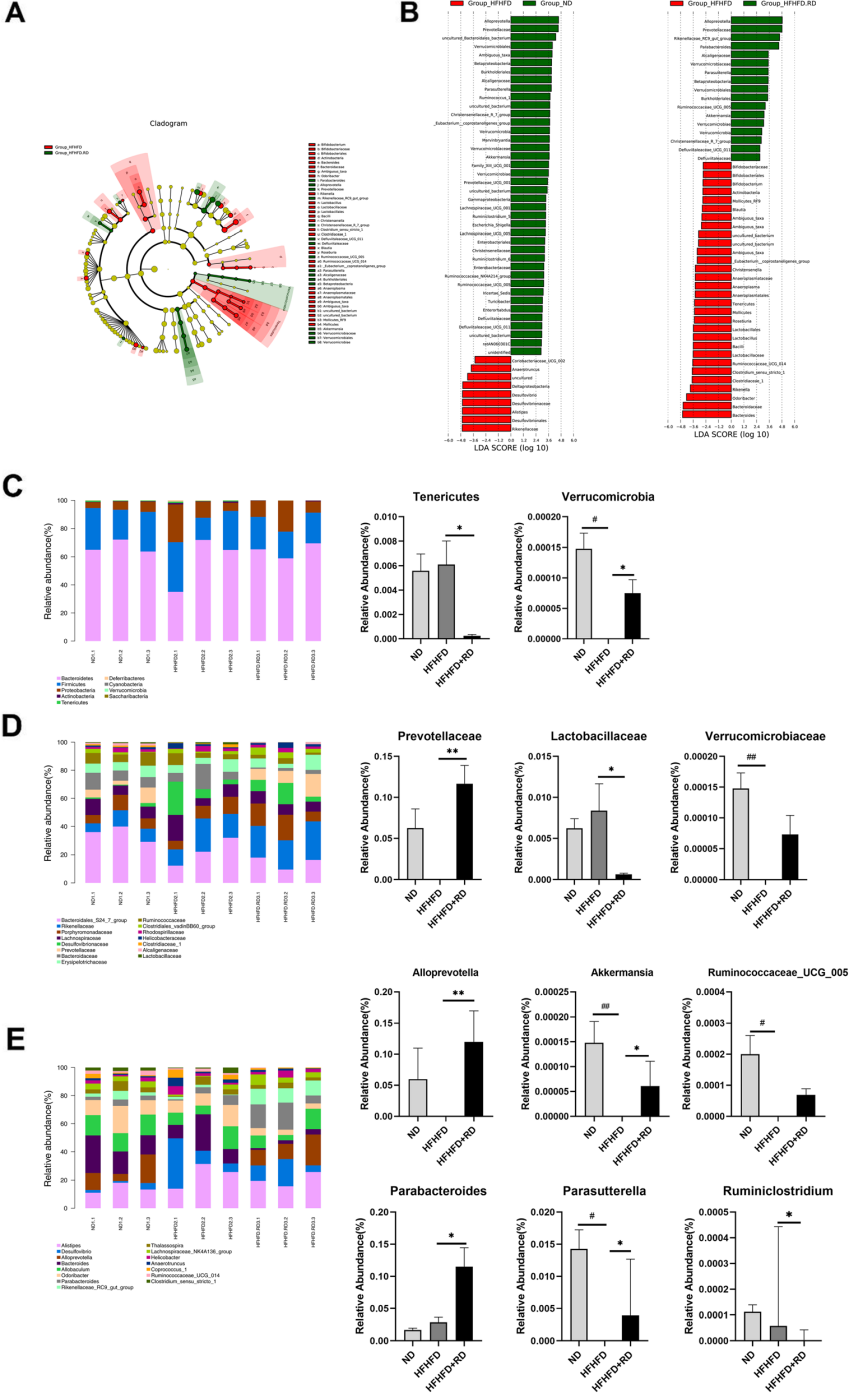
Gut microbiota composition analysis. **a** Cladogram generated from LEfSe analysis showing the relationship between taxon. **b** Linear discriminant analysis (LDA) scores derived from LEfSe analysis, showing the biomarker taxa LDA score of > 2 (the length of the bar represents the LDA score). **c**-**e** The composition of the gut microbiota in different classification levels. Data are expressed as the mean ± standard deviation or media with interquartile range. Differences were assessed by ANOVA or Kruskal-Wallis and denoted as follows, *P<0.05 vs. HFHFD group. #*P*<0.05 vs. ND group

To identify specific taxa related to resistant dextrin supplementation, relative abundance was assessed. At the phylum level, there was no significant differences in the ratio of Firmicutes/Bacteroidetes (Fig. S3). The relative abundance of Verrucomicrobia was notably higher compared to the HFHFD mice, and the relative abundance of Tenericutes was significantly decreased (Fig. 5c). At the family level, the results showed a lower level of Prevotellaceae and Verrucomicrobiaceae and a higher level of Lactobacillaceae in the HFHFD group. Conversely, these changes were restored in the mice of HFHFD+RD group (Fig. 5d). At the genus level, the HFHFD group presented a significantly lower level of Akkermansia, Alloprevotella, Parasutterella, Parabacteroides and Ruminococcaceae_UCG_005 but a higher level of Ruminiclostridium. While resistant dextrin treatment significantly restored the levels of the above bacteria to normal levels (Fig. 5e).

## Discussion

Resistant dextrin has been reported to improve insulin resistance and obesity. However, the molecular mechanisms underlying these effects remain unclear. In this study, we investigated the effects of resistant dextrin on HFHFD induced obesity mice. We found the beneficial effects of resistant dextrin on improving insulin resistance and reducing hepatic lipid deposition and these metabolic benefits of resistant dextrin may be associated with the modulation of gut microbiota.

As shown in our previous work, after 12 weeks of HFHFD feeding, mice exhibit obviously obesity and hyperglycemia [12]. In our current study, 10 weeks resistant dextrin intervention retarded the weight gain, which was under the circumstances of no change in food intake among three groups. Importantly, resistant dextrin remarkably improved hyperinsulinemia. This was consistent with the findings of previous studies showing that resistant dextrin can improve insulin resistance in overweight people or in those with type 2 diabetes [10, 11].

IRS-1-PI3K-AKT pathway is required for the maintenance of normal glucose metabolism. By activating this pathway, insulin promotes the utilization of hepatic glucose and reduces gluconeogenesis [13]. We studied the insulin signaling pathway in the liver, and found that the levels of p-AKT^ser473^, GLUT-2 proteins were decreased in the HFHFD group, and p-IRS-1^ser612^ was increased. As expected, resistant dextrin up-regulated the expressions of p-AKT^ser473^, GLUT-2 and reduced the phosphorylation of IRS-1 at ser612 site. Enhanced phosphorylation of the serine site of IRS-1 protein inhibits insulin signaling [14]. These results clearly showed that resistant dextrin successfully promoted insulin signaling pathway in the liver.

Hepatic lipid metabolism is closely linked with insulin resistance [15]. Our results showed that resistant dextrin improved serum lipid levels and reduced hepatic lipid deposition (Fig. 3). PPARɑ and FXR are crucial for fatty acid homeostasis [16, 17].Up-regulation of PPARα improves lipid metabolism [18]. Fxr−/− mice have elevated blood triglyceride and cholesterol levels [19]. In our study, we demonstrated that resistant dextrin could increase PPARα and FXR mRNA levels, as well as its target genes ACOX and CPT1α. We also demonstrated that resistant dextrin could increase the expression of PPARα and CPT1α at the protein levels. These results collectively suggest that resistant dextrin promoted fatty acid β-oxidation in the liver.

AMPK and Sirt1 play important roles in lipid homeostasis and insulin resistance. It has been reported that Sirt1 can stimulate the activation of AMPK, accelerating the decomposition of lipid to reducing liver fat accumulation [20]. Loss of liver Sirt1 leads to impaired PPARα signaling and decreased β-oxidation of fatty acids [21]. In addition, many studies have suggested that Sirt1 played a vital role in increasing insulin sensitivity. A study reported that Sirt1 agonists can improve glucose tolerance and insulin sensitivity in HFD mice [22]. It is well known that AMPK plays a central role in regulating insulin sensitivity, by reducing hepatic glucose production [23]. In our current study, we found that HFHFD obese mice treated with resistant dextrin expressed increased levels of Sirt1 and AMPK in liver. Thus, we suggest that resistant dextrin could effectively promote fatty acid β-oxidation and improve insulin sensitivity.

Dietary fiber can’t be hydrolyzed by digestive enzymes or be absorbed in the intestine, but can serve as a substrate for intestinal microbial fermentation [24], and many studies have indicated that the crucial roles of gut microbiota played in the host energy metabolism. Therefore, we employed 16 s rRNA sequencing to observe the effect of resistant dextrin on gut microbiota. PCoA analysis revealed a distinct separation in beta diversity of gut microbial communities among three groups. Besides, we analyzed the relative abundance of gut microbiota at different taxa levels. At the phylum level, Firmicutes and Bacteroidetes predominated in the majority. It is reported that the gut microbiota of obese rodent and human models display increased abundance of Firmicutes and decreased abundance of Bacteroidetes [25]. Our results showed that the Firmicutes/Bacteroidetes ratio was lower in mice treated with resistant dextrin group than that in the HFHFD group, which is positively correlated with obesity and diabetes. Interestingly, we also found that the relative abundance of Tenericutes was notably decreased by resistant dextrin supplementation, and the relative abundance of Verrucomicrobia, Verrucomicrobiaceae and Akkermansia were significantly higher compared to the HFFHD mice. Recent studies have reported that a reduction in the relative abundance of Tenericutes was detected in the inulin treated diabetic mice and rats [26, 27]. Everard et al. reported that Oligofructose increased the abundance of Akkermansia in the ob/ob mice and improved obesity-related phenotype [28], and Akkermansia is inversely correlated with metabolic disorders [29]. Supplementation of Akkermansia to HFD mice reduced obesity, decreased inflammatory markers, and improved insulin resistance [30].

Wu et al. suggested that dietary patterns affect gut microbial enterotypes. The long-term high-carbohydrate diet is characterized by Prevotella dominated enterotype, while the long-term high-protein and high-fat diet shows Bacteriodes enriched enterotype [31]. It has been reported that pectin-rich or whole grain diet enriches Prevotellaceae bacteria. Africa children with high fiber diet showed a significant enrichment in Prevotella, while European children who consume a typical low-fiber western diet lack this bacteria [32–34]. In our study, the HFHFD mice were dominated by Bacteriodes, whereas the ND mice were dominated by Allprevotella and Prevotellaceae. Importantly, mice with resistant dextrin supplementation also dominated by Allprevotella and Prevotellaceae, which is the same as the ND mice, indicating that resistant dextrin can switch HFHFD mice to Allprevotella and Prevotellaceae enterotype.

In addition, we found that resistant dextrin enhanced the relative abundance of Parasutterella and Parabacteroid, which were able to produce the SCFAs [35]. SCFAs were derived from the fermentation of non-digestible carbohydrates. Many studies have reported that they played a crucial role in the prevention and treatment of obesity-associated insulin resistance [36–38], and might via the upregulation of PPARα target genes, thereby increasing fatty acid oxidation [39]. This may help us to elucidate the mechanisms of the gut microbiota mediate the metabolic beneficial effects of resistant dextrin, though we didn’t detect the concentrations of SCFAs. Moreover, resistant dextrin increased Ruminococcaceae_UCG_005, which were beneficial to obesity and other associated metabolic disorders [40], while reduced Ruminiclostridium and Lactobacillaceae, which were increased in obesity [40]. Taken together, these results demonstrated that resistant dextrin can reduce HFHFD-induced gut microbiota dysbiosis.

## Conclusions

Our results demonstrated that resistant dextrin can significantly ameliorate liver insulin resistance induced by HFHFD, improve serum lipid levels, as well as reduce hepatic lipid deposition. Although further studies are required to define the mechanisms underlying gut microbiota mediated protective effects in insulin resistance, we speculate that the modulation of gut microbiota might be responsible for these beneficial effects of resistant dextrin, and our work may provide a key strategy to the management of type 2 diabetes.

## Supplementary information

**Additional file 1: Figure S1.** Body weight and FBG after 12 weeks of HFHFD feeding. **Figure S2.** The principal coordinates analysis (PCoA). **Figure S3.** The ratio of Firmicutes/Bacteroidetes in three groups.

## Data Availability

The data and materials used are available when requested.

## Abbreviations

T2DM: Type 2 diabetes mellitus
IR: Insulin resistance
FINS: Fasting insulin
TC: Total cholesterol
TG: Triglyceride
HDL-Ch: High-density lipoprotein cholesterol
LDL-Ch: Low-density lipoprotein cholesterol
SCFAs: Short chain fatty acids
FBG: Fasting blood glucose
GTT: Glucose tolerance test
ITT: Insulin tolerance test
FXR: Farnesoid X receptor
CPT1α: Carnitine palmitoyl transferase 1α
ACOX: Acyl-CoA oxidase
PPARα: Peroxisome proliferators-activated receptorα
AMPK: AMP activated protein kinase
IRS-1: Insulin receptor substrate-1
GLUT-2: Glucose transporter-2
RD: Resistant dextrin
