# Supplementary material for: Resistant dextrin improves high-fat-high-fructose diet induced insulin resistance
Source: Nutr Metab (Lond). 2020 May 15;17:36. doi: 10.1186/s12986-020-00450-2 (PMC7227367; doi:10.1186/s12986-020-00450-2)
Supplement: Supplementary file 1 — Additional file 1: Figure S1. Body weight and FBG after 12 weeks of HFHFD feeding. Figure S2. The principal coordinates analysis (PCoA). Figure S3. The ratio of Firmicutes/Bacteroidetes in three groups. [file 12986_2020_450_MOESM1_ESM.docx]

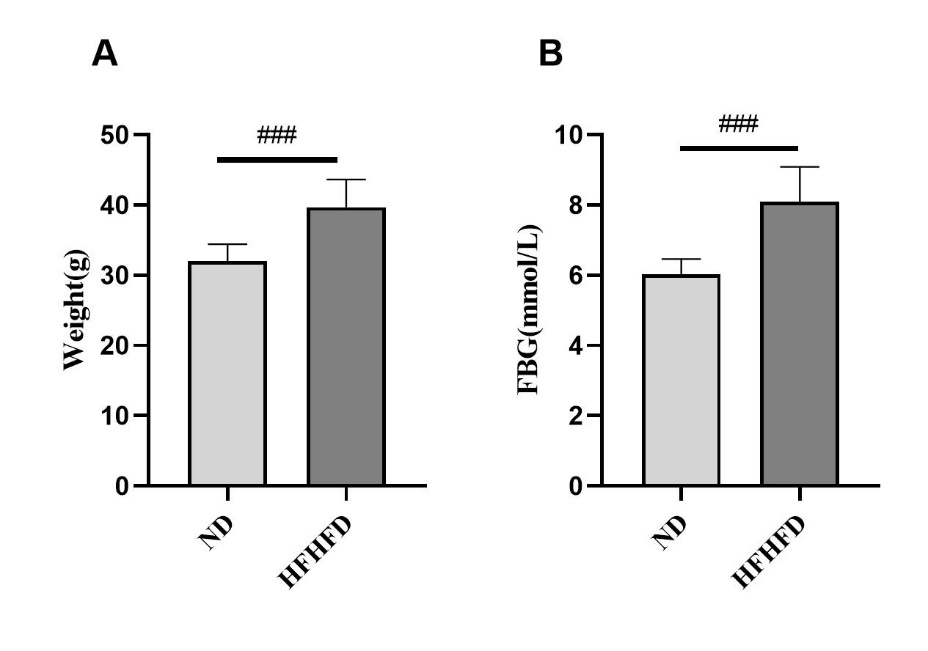


**Fig.S1** (A) Body weight and (B) fasting blood glucose (FBG) after 12 weeks of high-fat-high-fructose diet (HFHFD) feeding. ###*P*＜0.001 vs. ND group.


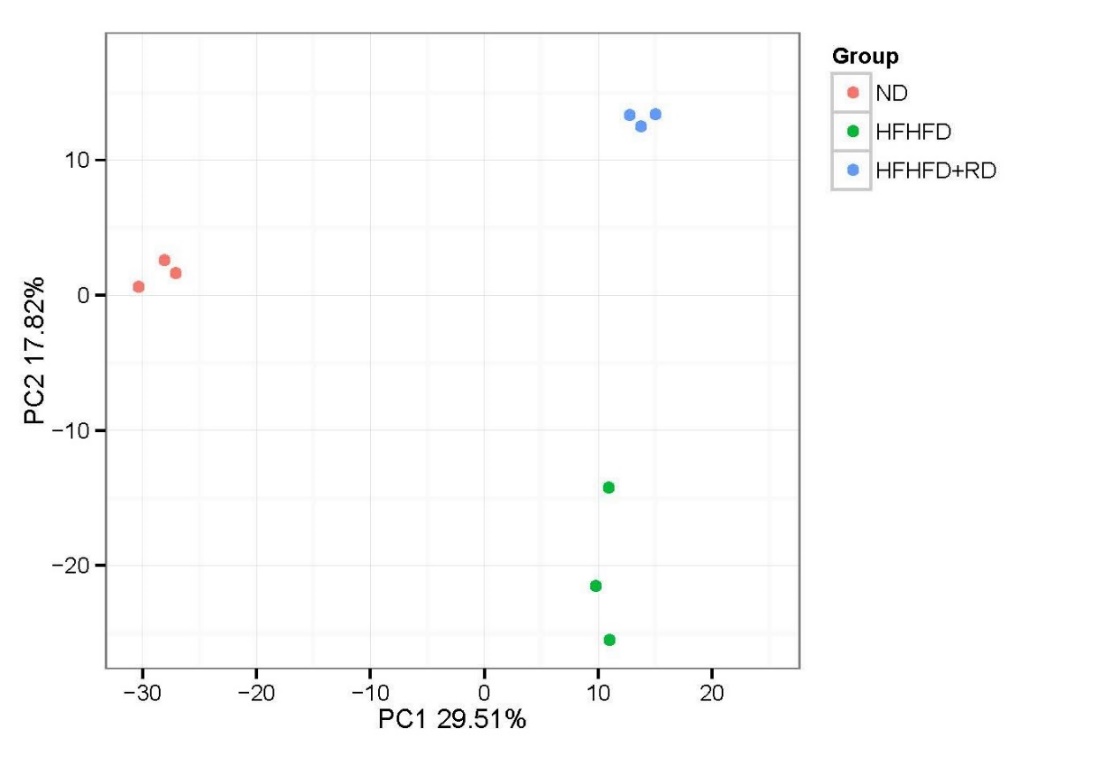


**Fig.S2** The principal coordinates analysis (PCoA).


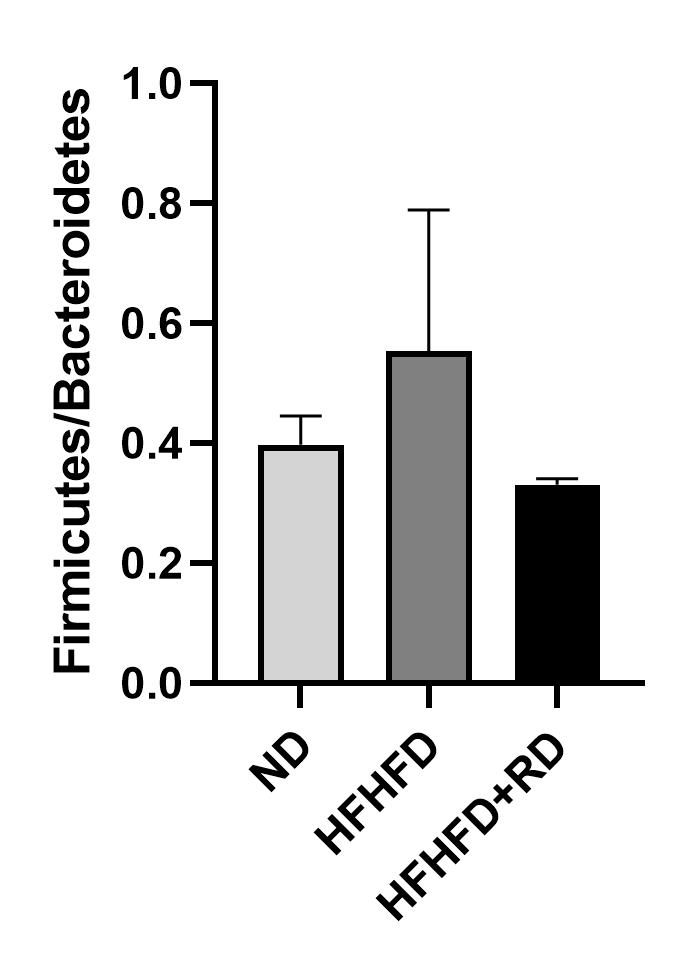


**Fig.S3** The ratio of Firmicutes/Bacteroidetes in three groups.
